# Involvement of Flagellin in Kin Recognition between Bacillus velezensis Strains

**DOI:** 10.1128/msystems.00778-22

**Published:** 2022-10-11

**Authors:** Yan Liu, Rong Huang, Yuqi Chen, Youzhi Miao, Polonca Štefanič, Ines Mandic-Mulec, Ruifu Zhang, Qirong Shen, Zhihui Xu

**Affiliations:** a Jiangsu Provincial Key Lab of Solid Organic Waste Utilization, Jiangsu Collaborative Innovation Center of Solid Organic Wastes, Nanjing Agricultural University, Nanjing, Jiangsu, Peoples R China; b Educational Ministry Engineering Center of Resource-Saving Fertilizers, The Key Laboratory of Plant Immunity, Nanjing Agricultural University, Nanjing, Jiangsu, Peoples R China; c Department of Microbiology, Biotechnical Faculty, University of Ljubljana, Ljubljana, Slovenia; University of California, Berkeley

**Keywords:** *Bacillus velezensis*, kin recognition, bacterial flagellum, variation of flagellin sequences, flagellin tertiary structure prediction, variation of hag gene

## Abstract

Kin discrimination in nature is an effective way for bacteria to stabilize population cooperation and maintain progeny benefits. However, so far, the research on kin discrimination for *Bacillus* still has concentrated on “attack and defense” between cells and diffusion-dependent molecular signals of quorum sensing, kin recognition in *Bacillus*, however, has not been reported. To determine whether flagellar is involve in the kin recognition of *Bacillus*, we constructed Bacillus velezensis SQR9 assembled with flagellin of its kin and non-kin strains, and performed a swarm boundary assay with SQR9, then analyzed sequence variation of flagellin and other flagellar structural proteins in B. velezensis genus. Our results showed that SQR9 assembled with flagellin of non-kin strains was more likely to form a border phenotype with wild-type strain SQR9 in swarm assay than that of kin strains, and that non-kin strains had greater variation in flagellin than kin strains. In B. velezensis, these variations in flagellin were prevalent and had evolved significantly faster than other flagellar structural proteins. Therefore, we proposed that flagellin is an effective tool partly involved in the kin recognition of B. velezensis strains.

**IMPORTANCE** Kin selection plays an important role in stabilizing population cooperation and maintaining the progeny benefits for bacteria in nature. However, to date, the role of flagellin in kin recognition in *Bacillus* has not been reported. By using rhizospheric Bacillus velezensis SQR9, we accomplished flagellin region interchange among its related strains, and show that flagellin acts as a mediator to distinguish kin from non-kin in B. velezensis. We demonstrated the polymorphism of flagellin in B. velezensis through alignment analysis of flagellin protein sequences. Therefore, it was proposed that flagellin was likely to be an effective tool for mediating kin recognition in B. velezensis.

## INTRODUCTION

In the natural environment, bacteria have a variety of multicellular cooperation lifestyles including biofilm (1), quorum sensing (2), swarming (3), sliding motility (4), production of extracellular enzymes (5), and labor division (6). It is advantageous for them to increase nutrient acquisition, resist unfavorable environments, avoid predation, and enhance the chance of survival and reproduction (7). A mechanism known as kin discrimination is used by bacteria to promote cooperative behavior among populations (8, 9). Kin discrimination means that bacteria treat organisms differently based on their kinship. They cooperate with organisms recognized as kin while competing with organisms recognized as non-kin (10–12). The 4 genes, *gyrA*, *rpoB*, *recA*, and *dnaJ*, have been used to determine the kinship of 39 Bacillus subtilis strains. In an analysis of the 4 genes, it was found that bacteria with similarities over 99.5% could be characterized as kin. The 2 kin strains merged on the swarm plate, while non-kin strains formed a boundary. In addition, kin strains can form a mixed biofilm on the root surface, but non-kin are not (13). Afterward, it was also discovered that the strains that do not produce surfactin could exploit the surfactin produced by the kin strains to restore part of the swarming motility ability, while the non-kin strains cannot provide this help (14). Kin discrimination is greatly important for bacteria to survive and development and fitness capabilities, but concrete research of molecular mechanisms is limited.

The research on bacterial kin discrimination mechanism is mostly focused on Gram-negative bacteria, such as Proteus mirabilis (15), Escherichia coli (16), Myxococcus xanthus (17, 18), and Pseudomonas aeruginosa (19); there are few studies on kin discrimination of Gram-positive bacteria, mainly including Staphylococcus aureus (20) and B. subtilis (21). In his review, Wall roughly summarizes the kin discrimination process of bacteria into 3 steps: receptor-ligand or receptor-receptor binding to recognition, signal or biochemical perception, and behavioral response (11). The research on kin discrimination for *Bacillus* has so far concentrated on “attack and defense” between cells, which is the second step of kinship discrimination. The kin discrimination system is highly complicated, involving genes encoding for proteins with a variety of functions, including microbial attack and defense genes: *wapAI*, *sdpABC*, *sdpIRs* and *skfA-H*, toxin, and immunity genes: *sunA* and *bacA*, and antibiotic attack-related genes: *lytST*, *yvrHB* and *sigW*, etc (10). The first step of kin recognition remains at the level of quorum sensing, which is a cell population behavior mediated by diffusion-dependent molecular signals (22), however, there is no report on whether there is a kin recognition mechanism in *Bacillus*.

Flagella is a complex structure regulated by a series of genes in bacteria (23). It is divided into 3 parts: trans-membrane basal body, hooks, and filaments, up to 15 μm in length (24). Flagella participates in both swimming and swarming motility, which are 2 important forms of bacteria movement (3, 25). Filaments are hollow tubes formed by the polymerization of flagellin monomers and are located outside the cell (26). Flagellin is divided into 4 domains: D0, D1, D2, and D3. The D0 and D1 domains are involved in the aggregation of flagellin monomers into flagellar filaments, which are highly conserved in various flagellated bacteria and are a microbe-associated molecular pattern. Flagellin D2 and D3 domains are not present in all flagellated bacteria, and their deletion weakens flagellin's intrinsic antigenicity but does not interfere with its immunostimulatory effects (27–29). Research has been conducted on the recognition and induction of various cells to bacterial flagella in recent years. For example, cells in vertebrates have evolved pattern recognition receptors, including TLR5 and NLRC4, which recognize the highly conserved region of flagellin as a danger signal (30). Plant cells recognition receptor FLS2, which can detect the 22 amino acids at the conserved N-terminal of flagellin, triggering a series of immune responses (31). A specific methanogenic archaeon perceived a bacterial flagellum protein and activated its methanogenesis, which suggests that the bacterium communicates with the archaeon by using its flagellum (32). However, the mechanism of bacteria-bacteria communication via flagella is poorly understood.

The filaments that extend outside the bacterial cell are several times longer than the cell body, and they might be used as a medium for mediating communication between bacteria, as the first step in kin discrimination. To date, it has not been reported whether flagellar are involved in bacterial recognition, or whether they are related to the evolution of strains of bacteria. In this study, a set of *hag* genes were exchanged to Bacillus velezensis with different phylogenetic distances from B. velezensis SQR9, to learn how differences in filament lead to a difference in recognition. In addition, we also performed an in-depth analysis of the sequence variation of flagellin and other flagellar structural proteins within B. velezensis species. The results showed that differences in flagellar filaments would alter the recognition phenotype of the strain on semi-solid plates, suggesting flagellin is involved in kin recognition of B. velezensis strains.

## RESULTS

### The lack of flagellin impairs kin recognition of B. velezensis SQR9.

**FIG 1:**
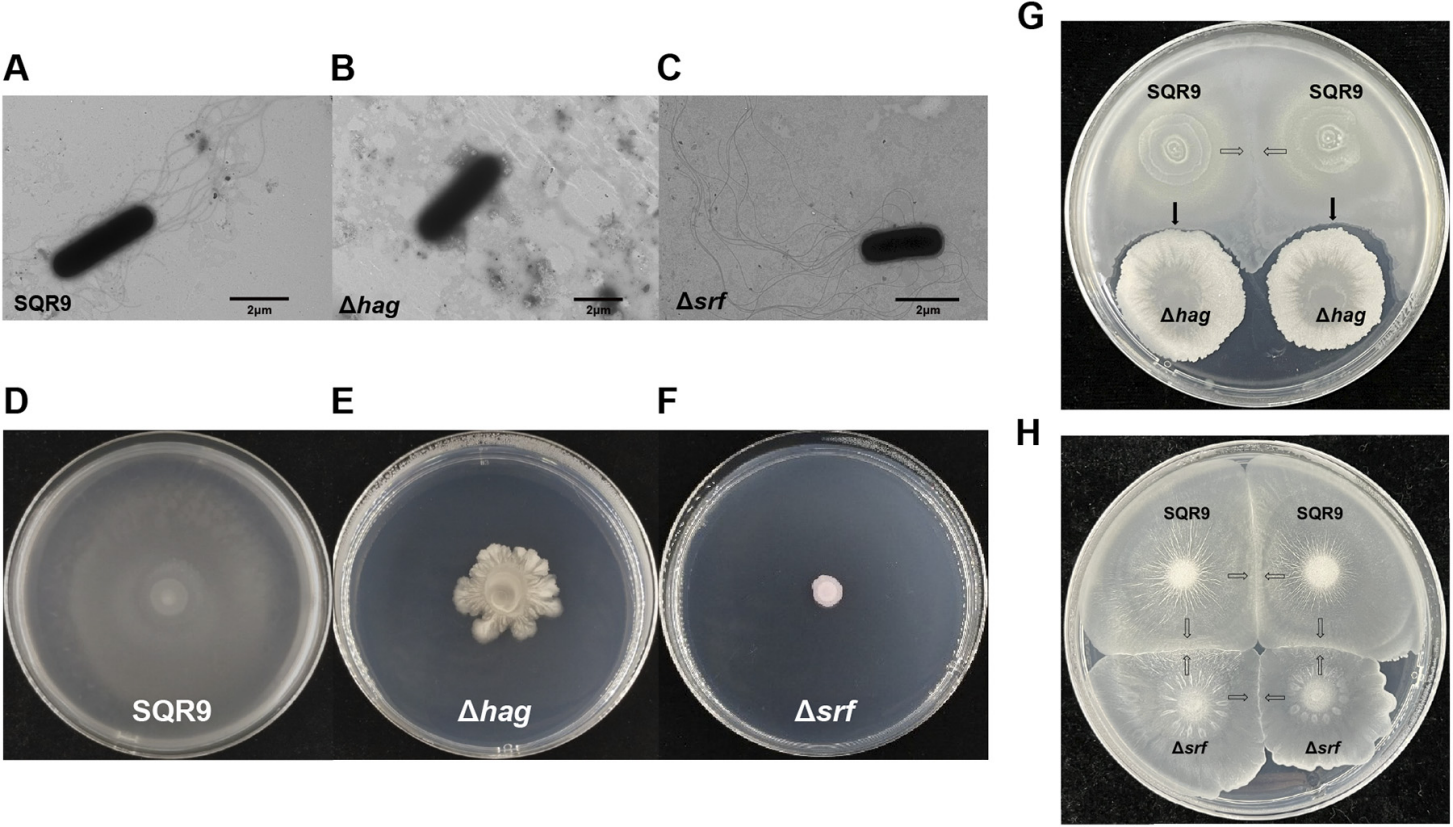
Deletion of *hag* gene in *B. velezensis* SQR9 resulted in the loss of flagella filaments and phenotype changes in the swarm boundary assay with itself. The transmission electron microscopy photos of the SQR9wt (A) and Δ*hag* mutant (B) showed that deletion of the flagellin *hag* gene prevented the strain from growing flagellar filaments, and deletion of the *srf* gene did not affect bacterial flagella assembly (C). The HT7700 transmission electron microscope (TEM) was operated at 80 kV to observe and photograph. The swarm phenotype of the SQR9wt (D), Δ*hag* (E), and Δ*srf* (F) displayed that the absence of flagellar filaments or surfactin renders the strain incapable of the swarm. (G) the loss of flagellar filaments hindered kin discrimination between SQR9wt and Δ*hag* in swarm assay. (H) Synthesis defect of surfactin did not affect kin discrimination between SQR9wt and Δ*srf* in swarm assay. The white arrows represented merge phenotype, while the black arrows represented boundary phenotype. All results are representative of three experiments.

The flagella are very important motility organs for the bacterium, and the bacteria without flagella are not able to swim and swarm (3, 25). B. velezensis SQR9 is a plant-growth promoting rhizobacteria (PGPR) strain, isolated from cucumber rhizosphere soil. B. velezensis SQR9 mutant strain Δ*hag* lacked flagellar filaments and did not swarm on a Semi-solid medium (Fig. 1A, B, D, and E). The strain SQR9wt (wild type) merged with itself on the swarming plate (Fig. 1G, white arrows), while cannot merge with mutant Δ*hag* without flagellar filaments, and even it avoided the surrounding area of Δ*hag* to grow (Fig. 1G, black arrows). Moreover, we used the Δ*srf* (the surfactin synthetic gene mutant) as a control with motility mutants different than Δ*hag*. Results showed that Δ*srf* mutant of B. velezensis SQR9 has the complete and normal flagella as the wild-type strain (Fig. 1C), but loses its swarming ability (Fig. 1F). When the Δ*srf* mutant meets the SQR9 wild-type during swarm assay, they merged on the swarm plate (Fig. 1H). These results indicated that flagellin is potentially involved in kin recognition for B. velezensis SQR9. Additionally, we observed that the Δ*srf* mutant with impaired swarming ability could exploit surfactin from wild-type as biosurfactant to restore its swarming ability (Fig. 1H), and these observations were consistent with the formerly report by Nicholas A. Lyons and Roberto kolter (33). Based on the results, kin recognition and cooperation were hindered between the mutant strain and the wild-type strain, suggesting that bacterial flagellar filaments might play a role in kin recognition in B. velezensis.

### B. velezensis SQR9 assembled with “non-kin” flagella now behaved as non-kin when confronted with the wild-type in swarming assay.

**FIG 2:**
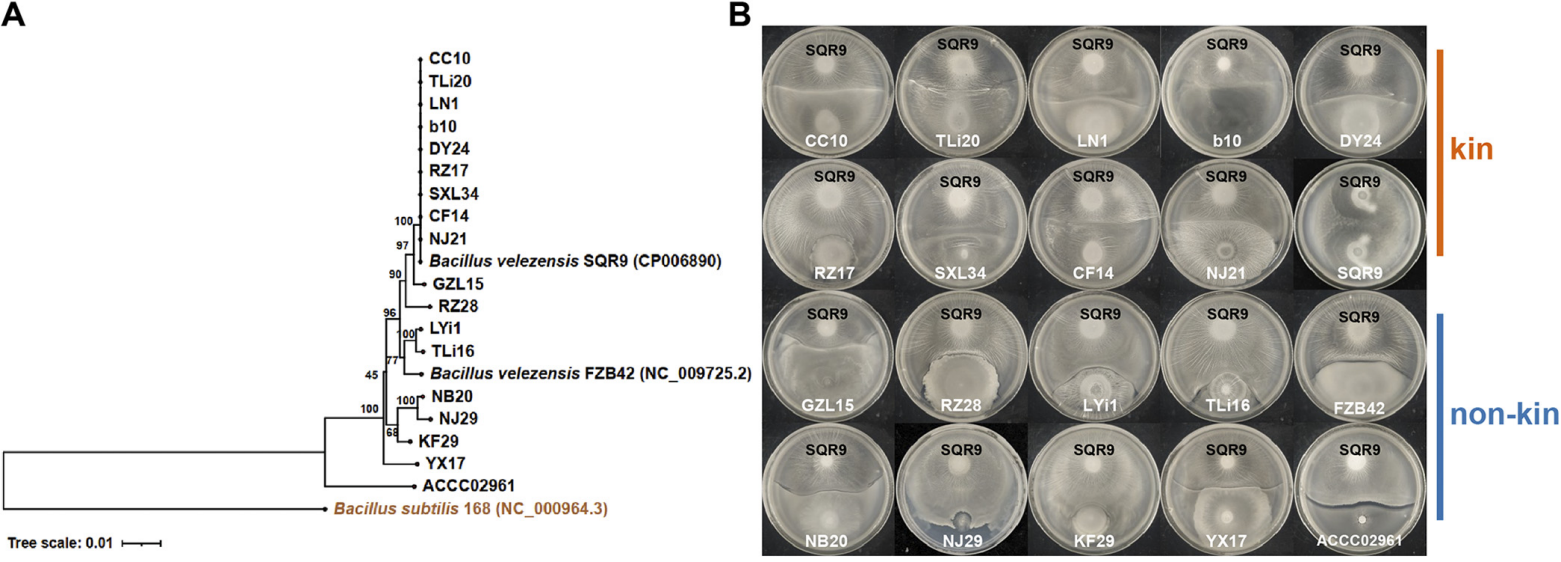
The recognition phenotype of *B. velezensis* SQR9 and its relative on swarm plate varied from merging to the boundary with their phylogenetic distance. (A) The tree was constructed on *gyrA* gene sequences (2264 bp) using MEGA (v.5.05) for Neighbor-Joining, the B. subtilis 168 (NC_000964.3) was selected as the outgroup. And the reliability of clades was tested by the 1000 bootstrap replications. (B) displayed swarm phenotype of *B. velezensis* SQR9 and 20 strains, which were sorted according to the phylogenetic distance between 20 strains and SQR9 on the tree from near to far. The first two rows were the kin of SQR9, which merged with SQR9; the last two rows were the non-kin of SQR9, which formed a boundary with SQR9, and the boundary tends to widen with the increase of the phylogenetic distance between the non-kin strains and SQR9. The results are representative of three experiments.

To study how flagellar filaments affect kin recognition ability in B. velezensis, we collected 20 strains belonging to B. velezensis, constructed a phylogenetic tree, and performed a swarm boundary assay between SQR9 and other 19 strains, to characterize kinship distance between 20 strains (Fig. 2). The phylogenetic tree was constructed basing the housekeeping gene *gyrA* (2264 bp), and 9 strains had the same *gyrA* gene sequences as SQR9 and on a phylogenetic tree branch, the other 10 strains had different *gyrA* gene sequences from SQR9 and were located on different tree branches (Fig. 2A). 9 strains on the same branch with SQR9 formed merge with SQR9 on swarming plates (defined as kin), while 10 strains on different branches without SQR9 formed boundary phenotype with SQR9 (defined as non-kin) (Fig. 2B). Interestingly, among the non-kin strains and SQR9, the boundary width was positively correlated with kinship distance (Fig. S1).

10.1128/msystems.00778-22.1 FIG S1 The swarm boundary width of SQR9 and non-kin strains was positively correlated with their phylogenetic distance. (A) The tree was constructed on *gyrA* gene sequences (2264bp) using MEGA (v.5.05) for Neighbor-Joining, the B. subtilis 168 (NC_000964.3) was selected as the outgroup. And the reliability of clades was tested by the 1000 bootstrap replications. (B) The swarm boundary width of SQR9 and 10 non-kin strains from the phylogenetic distance. Each pair of strains included 9 replicates of the swarm boundary assay phenotype, and each replicate was measured three times using the Image J (v.1.53c). The point plots were drawn using the ggplot2 package and linear regression analysis is performed using the lm() function in R (v.4.0.3). Download FIG S1, JPG file, 0.4 MB. Copyright © 2022 Liu et al. 2022 Liu et al. https://creativecommons.org/licenses/by/4.0/ This content is distributed under the terms of the Creative Commons Attribution 4.0 International license.

**FIG 3:**
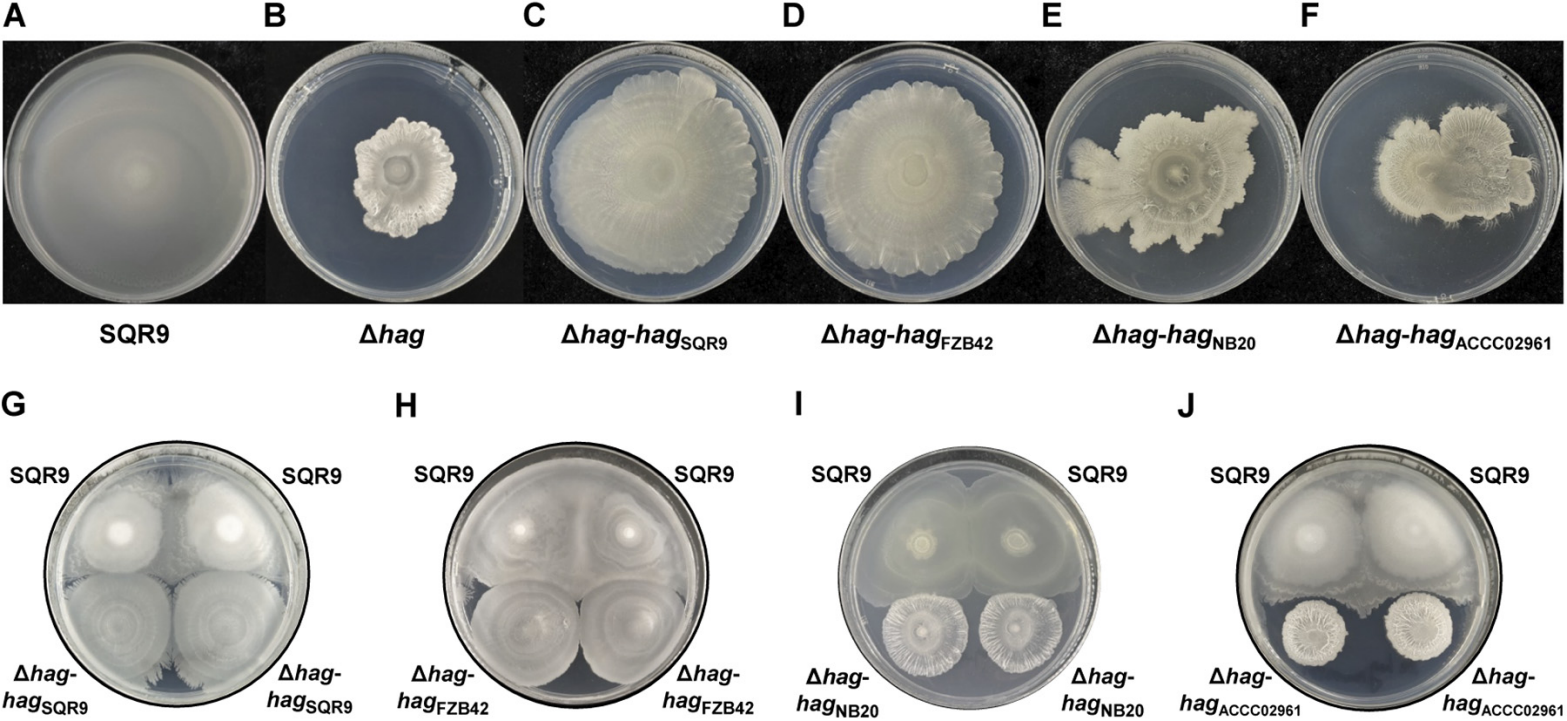
Wild-type strain SQR9 and its relatives (Δ*hag* mutant of SQR9 assembled with flagellin of kin and non-kin strains) on swarm plates (0.7% gar) show different recognition phenotypes. (A) to (F) The farther the phylogenetic distance between the source strains of flagellin complemented and SQR9, the more difficult it is to restore the swarm ability of flagellin mutant Δ*hag*. The swarm phenotypes of single strain were displayed, including SQR9wt (A), Δ*hag* (flagellin mutant of SQR9) (B), Δ*hag*-*hag*_SQR9_ (Δ*hag* carrying *hag* gene of kin strain SQR9) (C), Δ*hag*-*hag*_FZB42_ (Δ*hag* carrying *hag* gene of non-kin strain FZB42) (D), Δ*hag*-*hag*_NB20_ (Δ*hag* carrying *hag* gene of non-kin strain NB20) (E) and Δ*hag*-*hag*_ACCC02961_ (Δ*hag* carrying *hag* gene of non-kin strain ACCC02961) (F). The *hag* genes were complemented into Δ*hag* using plasmids. (G–J) The farther the phylogenetic distance between the source strains of flagellin complemented and SQR9, the more similar the swarming phenotype of SQR9 carrying heterologous flagellin and SQR9wt to that of SQR9wt and Δ*hag*. The swarm phenotypes of two strains were shown, including SQR9wt and Δ*hag*-*hag*_SQR9_ (G), SQR9wt and Δ*hag*-*hag*_FZB42_ (H), SQR9wt and Δ*hag*-*hag*_NB20_ (I), SQR9wt and Δ*hag*-*hag*_ACCC02961_ (J). The pictures of the plates were acquired 48 h after inoculation, and the results are representative of three experiments.

After knowing the kinship of those 20 B. velezensis strains, 4 strains with different kinship distances to strain SQR9 were selected: SQR9, FZB42, NB20, and ACCC02961. These 4 strains all possessed complete pericyte flagella and superior swarm motility (Fig. S2A, G-I), and were all merged with themselves on swarming plates (Fig. S3E-H). Four plasmids containing *hag* genes amplified from genome DNA of these 4 strains were transformed into mutant Δ*hag* of SQR9, respectively. The Δ*hag* carrying corresponding plasmids were respectively named Δ*hag*-*hag*_SQR9_, Δ*hag*-*hag*_FZB42_, Δ*hag*-*hag*_NB20_, and Δ*hag*-*hag*_ACCC02961_. Transmission electron microscope (TEM) images showed that the flagellar filaments were recovered after the transformation of the plasmid, which contains a different *hag* gene (Fig. S2C-F). Interestingly, the Δ*hag*-*hag*_SQR9_, Δ*hag*-*hag*_FZB42_, Δ*hag*-*hag*_NB20_, and Δ*hag*-*hag*_ACCC02961_ restored a certain motility ability on semi-solid plates, however, the first 2 strains recovered considerably more than the latter 2 on swarm assay medium with 0.5% and 0.7% agar (Fig. 3A to F and Fig. S4). In swarm boundary assay, Δ*hag*-*hag*_SQR9_ and Δ*hag*-*hag*_FZB42_ could merge with SQR9wt (Fig. 3G and H and Fig. S3J and K), Δ*hag*-*hag*_NB20_ was an intermediate phenotype with SQR9wt (Fig. 3I and Fig. S3L), Δ*hag*-*hag*_ACCC02961_ even formed boundary phenotype with SQR9wt (Fig. 3J and Fig. S3M). In summary, when Δ*hag* mutant of SQR9 was complemented different flagellar filaments from kin and non-kin strains, the swarm phenotypes against wild-type SQR9 are similar to donor strains of *hag* gene.

10.1128/msystems.00778-22.2 FIG S2 The flagellin mutant Δ*hag* complemented with the *hag* gene of kin and non-kin strains restored flagellar filaments growth. The transmission electron microscopy photos of strain were displayed, including SQR9wt (A), Δ*hag* (flagellin mutants of SQR9) (B), Δ*hag*-*hag*_SQR9_ (Δ*hag* carrying *hag* gene of kin strain SQR9) (C), Δ*hag*-*hag*_FZB42_ (Δ*hag* carrying *hag* gene of non-kin strain FZB42) (D), Δ*hag*-*hag*_NB20_ (Δ*hag* carrying *hag* gene of non-kin strain NB20) (E), Δ*hag*-*hag*_ACCC02961_ (Δ*hag* carrying *hag* gene of non-kin strain ACCC02961) (F), FZB42wt (G), NB20wt (H) and ACCC02961wt (I). The HT7700 transmission electron microscope (TEM) was operated at 80 kV to observe and photograph. Download FIG S2, JPG file, 0.9 MB. Copyright © 2022 Liu et al. 2022 Liu et al. https://creativecommons.org/licenses/by/4.0/ This content is distributed under the terms of the Creative Commons Attribution 4.0 International license.

10.1128/msystems.00778-22.3 FIG S3 Swarm ability of strains SQR9wt (A), FZB42wt (B), NB20wt (C) and ACCC02961wt (D) on swarm plate (0.7% agar). The merging phenotype of strains SQR9wt (E), FZB42wt (F), NB20wt (G) and ACCC02961wt (H) with itself on swarm plate (0.7% agar). The merging phenotype of Δ*hag* mutant (I) and SQR9 carrying heterologous flagellin (Δ*hag*-*hag*_SQR9_ [J], Δ*hag*-*hag*_FZB42_ [K], Δ*hag*-*hag*_NB20_ [L], Δ*hag*-*hag*_ACCC02961_ [M]) with itself on swarm plate (0.7% agar). The pictures of the plates were acquired 48h after inoculation, and the results are representative of 3 experiments. Download FIG S3, JPG file, 1.1 MB. Copyright © 2022 Liu et al. 2022 Liu et al. https://creativecommons.org/licenses/by/4.0/ This content is distributed under the terms of the Creative Commons Attribution 4.0 International license.

10.1128/msystems.00778-22.4 FIG S4 The flagellin mutant Δ*hag* complemented with the *hag* gene of kin and non-kin strains restored motility ability. The motility ability of strains was tested using a B medium with 0.3%, 0.5%, and 0.7% agar. The halo area of strains was measured in Image J (v.1.53c) after 24 h of inoculation. The results are representative of three experiments. The bars with different letters are statistically different according to the two-sided Duncan’s multiple range tests (*P < *0.05). Download FIG S4, JPG file, 0.4 MB. Copyright © 2022 Liu et al. 2022 Liu et al. https://creativecommons.org/licenses/by/4.0/ This content is distributed under the terms of the Creative Commons Attribution 4.0 International license.

### Production of bacillunoic acid by Δ*hag* mutant and its flagellin gene complementary strains is similar to SQR9 wild type.

**FIG 4:**
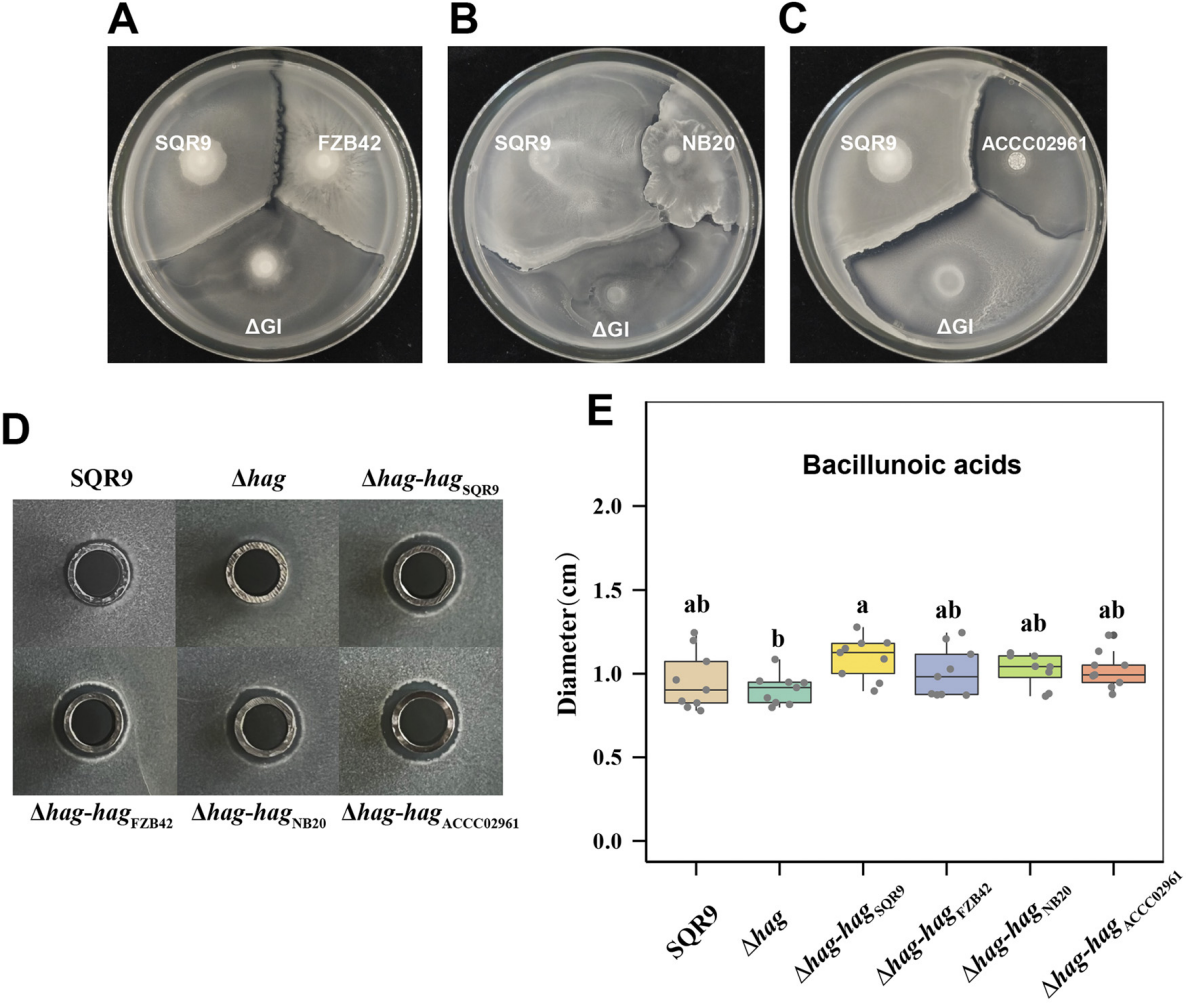
The deletion and complementation of the *hag* gene of SQR9 had no significant effect on the production of antibacterial substances Bacillunoic acids. Compared with SQR9wt, the ΔGI mutant (the bacillunoic acid synthetic gene mutant) formed a weakened boundary phenotype with non-kin strains FZB42 (A), NB20 (B) and ACCC02961 (C). The ΔGI is a deletion mutant of the gene island in strain SQR9 for the synthesis of bacillunoic acids. The pictures of the plates were acquired 48 h after inoculation, and the results are representative of three experiments. (D) The photo of the antagonism circle assay of *B. velezensis* FZB42 by the fermentation supernatant of strains SQR9wt, Δ*hag*, and four *hag* gene complemented strains (Δ*hag*-*hag*_SQR9_, Δ*hag*-*hag*_FZB42_, Δ*hag*-*hag*_NB20_ and Δ*hag*-*hag*_ACCC02961_). (E) The production of Bacillunoic acids in SQR9 wild-type, Δ*hag* mutants, Δ*hag*-*hag*_SQR9_, Δ*hag*-*hag*_FZB42_, Δ*hag*-*hag*_NB20_ and Δ*hag*-*hag*_ACCC02961_, was assessed using the antagonism assay of the fermentation supernatant of the tested strain to *B. velezensis* FZB42. The antagonism assays for each strain included nine replicates. The box plots were drawn using R (v.4.0.3), and the analysis of significant differences was performed using Duncan’s multiple range tests (*P* < 0.05) on SPSS (v. 25).

We demonstrated in previous studies that changes in flagella (deletion or replacement with non-kin flagella) resulted in a change in the recognition phenotype (from merge to boundary) of the mutant and complemented strains with SQR9wt on swarm plate. Whether this phenomenon is caused by changes in the flagella or by changes in the secretion of antibiotics needs to be further explored. Our previous work shows that B. velezensis SQR9 can secrete a variety of antibacterials, including 3 lipopeptides bacillomycin D, fengycin and bacillibactin; three polyketides bacillaene, difficidin, and macrolactin (34, 35). Moreover, a novel antimicrobial fatty acid, named Bacillunoic acids, which showed strong antibacterial against closely related *Bacillus* strains (36). In our preliminary experiments, strains of 6 mutants: Δ*bmy* (synthetic gene mutant of bacillomycin D), Δ*fen* (synthetic gene mutant of fengycin), Δ*dhb* (synthetic gene mutant of bacillibactin), Δ*bae* (synthetic gene mutant of bacillaene), Δ*dfn* (synthetic gene mutant of difficidin), and Δ*mln* (synthetic gene mutant of macrolactin), both merged with SQR9wt (Fig. S5A to F) and showed similar boundary phenotype with non-kin strains ACCC02961 as SQR9wt strain (Fig. S5H to M). Only the ΔGI mutant (the bacillunoic acid synthetic gene mutant) formed boundary phenotype with SQR9wt (Fig. S5G) and formed a weakened boundary phenotype with non-kin strains FZB42, NB20 and ACCC02961 (Fig. 4A to C), indicated that bacillunoic acid is partly involved in the kin discrimination of SQR9.

10.1128/msystems.00778-22.5 FIG S5 Only the secondary metabolite bacillunoic acid was involved in kin discrimination of *B. velezensis* SQR9. The swarm boundary assays: (A) SQR9 and Δ*bmy* (synthetic gene mutants of bacillomycin D); (B) SQR9 and Δ*fen* (synthetic gene mutants of fengycin); (C) SQR9 and Δ*dhb* (synthetic gene mutants of bacillibactin); (D) SQR9 and Δ*bae* (synthetic gene mutants of bacillaene); (E) SQR9 and Δ*dfn* (synthetic gene mutants of difficidin); (F) SQR9 and Δ*mln* (synthetic gene mutants of macrolactin); (G) SQR9 and ΔGI (synthetic gene mutants of bacillunoic acid); (H) SQR9, ACCC02961 and Δ*bmy*; (I) SQR9, ACCC02961 and Δ*fen*; (J) SQR9, ACCC02961 and Δ*dhb*; (K) SQR9, ACCC02961 and Δ*bae*; (L) SQR9, ACCC02961 and Δ*dfn*; (M) SQR9, ACCC02961 and Δ*mln*; (N) SQR9, ACCC02961 and ΔGI. Download FIG S5, JPG file, 1.2 MB. Copyright © 2022 Liu et al. 2022 Liu et al. https://creativecommons.org/licenses/by/4.0/ This content is distributed under the terms of the Creative Commons Attribution 4.0 International license.

The bacillunoic acids secreted by SQR9 into the fermentation supernatant can effectively antagonize B. velezensis FZB42, making it form an antagonistic circle around the Oxford cup with the fermentation supernatant. We compared the production of bacillunoic acids by using the method of the antagonistic circle diameter measurement (Fig. 4D). Results showed that deletion of flagella or complementation for the various *hag* genes in SQR9 does not affect bacillunoic acid production (Fig. 4E). In addition, we tested the surfactin production of SQR9wt and Δ*hag* mutant in liquid culture using high-performance liquid chromatography. Again, similar production of surfactin was observed between the 2 strains (Fig. S6). These results are consistent with our conclusion that flagellin is partly involved in kin recognition in B. velezensis SQR9 without affecting the antibacterial compounds production.

10.1128/msystems.00778-22.6 FIG S6 The deletion of the *hag* gene of SQR9 had no significant effect on the production of antibacterial substances surfactin. Chromatographic peaks of surfactin standard (A) and SQR9wt (B) and Δ*hag* mutant (C) fermentation supernatant. The peaks pointed to by the black arrows are the peaks of the 3 homologs of surfactin of interest in this study. (D) The production of surfactin in SQR9 wild-type and Δ*hag* mutants was assessed using high-performance liquid chromatography (HPLC). The peak areas shown are the sum of the chromatographic peak areas of the 3 surfactin homologs. Each strain sample was tested three times, and the analysis of significant differences was performed using the independent sample T-Test (*P* < 0.05) on SPSS (v. 25). Download FIG S6, JPG file, 0.7 MB. Copyright © 2022 Liu et al. 2022 Liu et al. https://creativecommons.org/licenses/by/4.0/ This content is distributed under the terms of the Creative Commons Attribution 4.0 International license.

### For B. velezensis, the flagellin heterogeneity of non-kin strains is much higher than kin.

**FIG 5:**
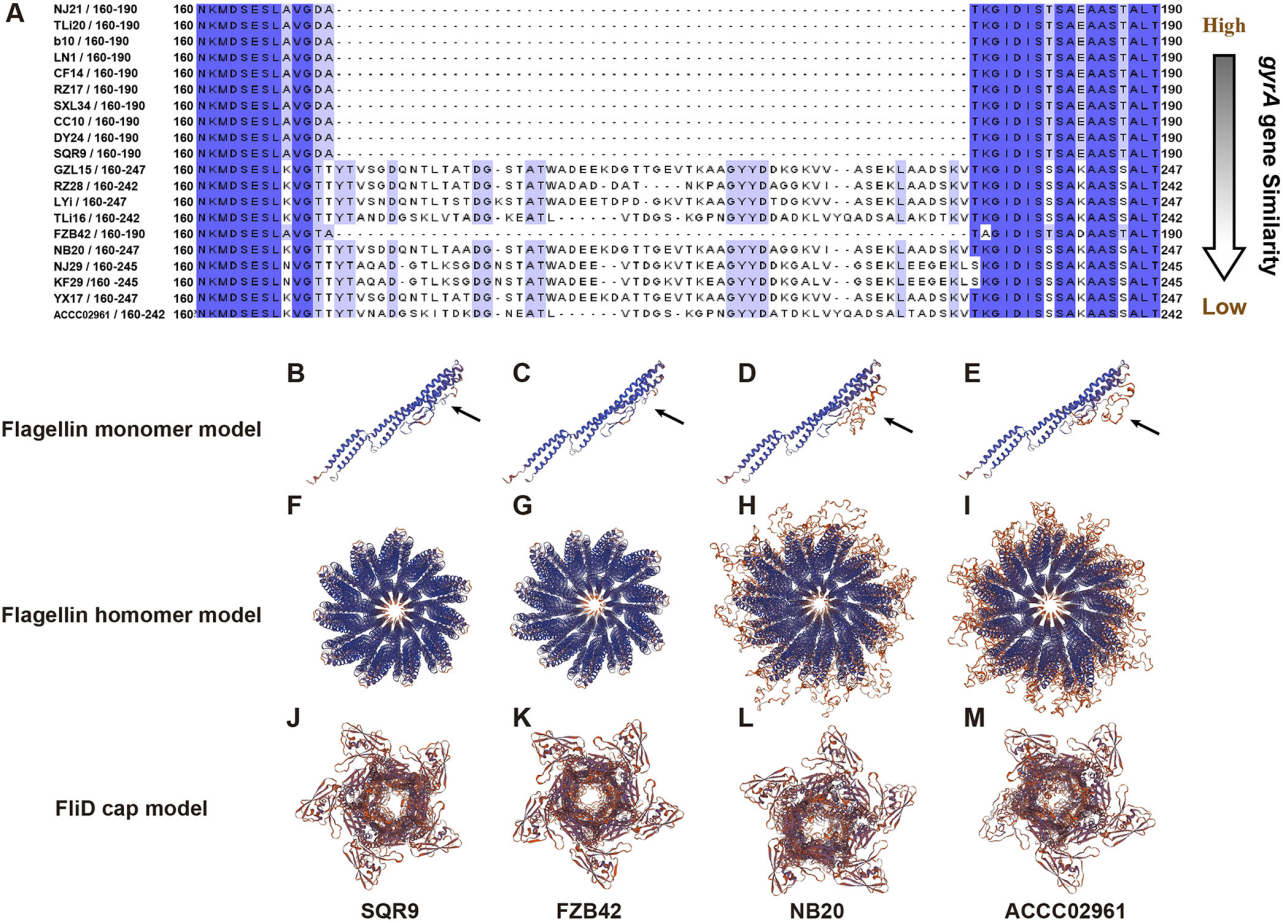
Flagellin sequence and protein structure varied more between non-kin strains than between kin strains in *B. velezensis*. These pictures showed the variable region of the *flagellin sequences* of 20 *B. velezensis* strains (A) and the protein structure prediction of flagellar monomer (B) to (E), flagellar homomer (F) to (I), and flagellar cap (J) to (M) according to four *B. velezensis* strains: SQR9, FZB42, NB20, and ACCC02961. These prediction structures of flagellin homomer and flagellar cap showed their cross sections from the top view of the filaments. The *hag* gene sequences were aligned using the L-INS-I method of MAFFT (v7.487) and displayed using Jalview (v.2.11.1.5), and the protein structure of flagellin was predicted on the Swiss-Model website.

The results above suggest that flagellin in B. velezensis strains might be involved in the recognition of kin, we next want to investigate whether the structure of flagellin is related to the kinship. We sequenced the *hag* gene sequences of the 20 B. velezensis strains and translated them into protein sequences, then analyzed their structural features. Results showed that both ends of the sequences were relatively conservative, and there were only several variant bases, but the middle part of the sequences was very different (Fig. S7). To observe the details of the variable region of the flagellin sequences more clearly, we cut and display the variable region separately (Fig. 5A). The sequences belonging to different strains were sorted according to the *gyrA* gene similarity of the strains and SQR9, that was, the strain ACCC02961 at the bottom had the farthest kinship with SQR9. The strains on a tree branch with SQR9 have the same flagellin sequence as SQR9. Other strains had an extra sequence in the variable region of the flagellin sequence (the length of sequences was 52–57 aa), except for strain FZB42 (Fig. 2A and Fig. 5A). The flagellin sequence variant region of FZB42 was very similar to SQR9, compare to strains on adjacent branches (Fig. 5A). This may be due to individual differences in strains, or the evolutionary rate of the *hag* gene was not strictly consistent with the *gyrA* gene.

10.1128/msystems.00778-22.7 FIG S7 The conserved and variable regions of the flagellin sequences from 20 *B. velezensis* strains. There was a lot of variation in the central region of sequences, and in addition to mutations at individual sites, there were many large insertions and deletions. their lengths were in the range 266-324 aa and were aligned using the L-INS-I method of MAFFT (v7.487) and displayed using Jalview (v.2.11.1.5). Download FIG S7, JPG file, 2.8 MB. Copyright © 2022 Liu et al. 2022 Liu et al. https://creativecommons.org/licenses/by/4.0/ This content is distributed under the terms of the Creative Commons Attribution 4.0 International license.

To clarify what difference the variable region of the flagellin sequence caused, we selected 4 strains that were known genomes: SQR9, FZB42, NB20, and ACCC02961, then applied the entire *hag* gene sequences to predict the tertiary structure of flagellin and flagellar filaments by using comparative modeling method based on Swiss-model database. The most similar template matched by the flagellin sequences of four strains was the same template 6t17.1.A, and the details of template matching information were placed in Table S1 (see Table S1 at [https://zenodo.org/record/7131344#.Yzei8thBxPY]). Based on the prediction results of the flagellin monomer, the variable region of the flagellin monomers of the NB20 and ACCC02961, which had more β-strands and random coils than that of SQR9 and FZB42 (Fig. 5B to E). After the flagellin monomers were assembled into flagellar filaments, the part of β-strands and random coils was exposed on the periphery of the columnar flagellar filaments (Fig. 5F to I). In addition, we also carried out the protein structure prediction of the flagellar cap structure (Table S1; at [https://zenodo.org/record/7131344#.Yzei8thBxPY]) and found that the flagellar cap structure of the 4 strains was very similar, and they were all composed of 5 protein monomers (Fig. 5J to M). The flagellar cap was first assembled on the flagella, and then helps and regulates the flagellin monomers to gradually assemble into flagellar filaments (37, 38). Therefore, SQR9 can assemble different flagellin monomers into flagellar filaments. Taken together, these results above suggest that flagellin in B. velezensis strains might be involved in the recognition of kin.

### The variation of flagellin is higher than other flagellar proteins, and it is not conservative in B. velezensis.

In the above analysis, we observed a wider variation in a specific area of the *hag* gene among 20 B. velezensis strains, we next want to explore the flagellin diversity of B. velezensis. Therefore, we analyzed the sequence variation of flagellin in 190 B. velezensis strains from the NCBI genome database and used several other flagellar structural proteins as a reference.

**FIG 6:**
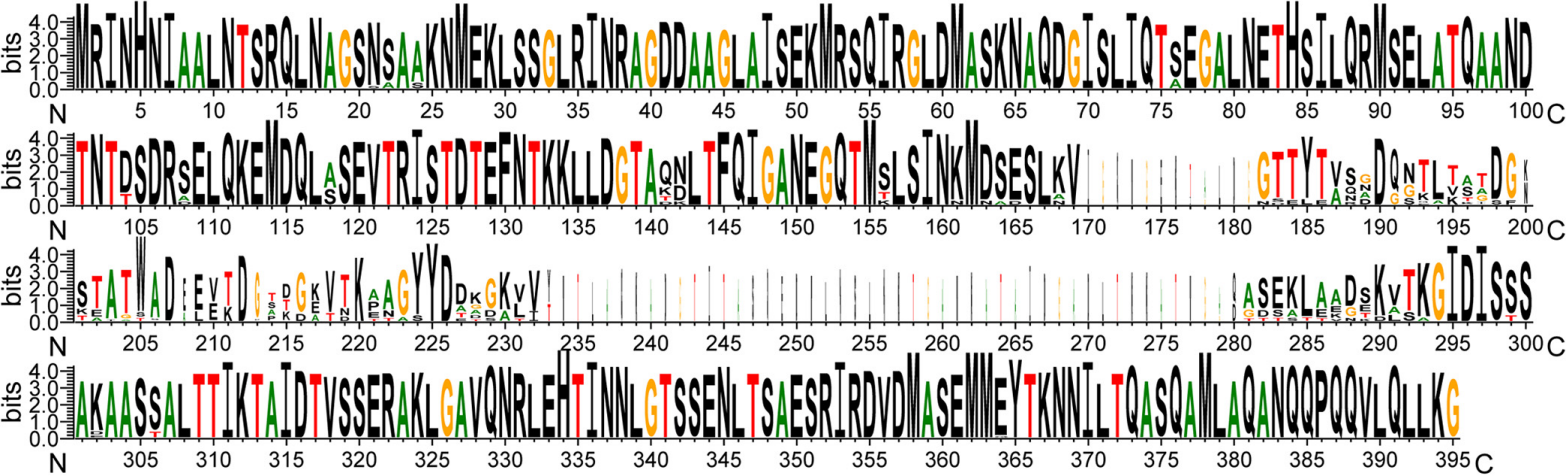
Flagellin sequences logos exhibit dramatic variation in *B. velezensis*. The variation was mainly concentrated in the 170–293 sites region of flagellin sequences, which contained many large insertions and deletions. These sequences came from 190 *B. velezensis* strains in the NCBI database, their lengths were in the range of 276–384 amino acids, and were aligned using the L-INS-I method of MAFFT (v7.487) and displayed using WebLogo (v.3.7.4).

During alignment, it was found that the middle region of the flagellin sequences (170-293 sites) showed the most variation (Fig. 6), with many large insertions or deletions, roughly divided into 3 lengths: two amino acids, 21 amino acids, and 110 amino acids (Fig. S9; at [https://zenodo.org/record/7086025#.YyR7PaRBxPY]). The divergence of the flagellin sequences was greater than that of the 20 B. velezensis strains above (Fig. S7). In contrast with the intermediate variable region of flagellin, both ends of flagellin have fairly conservative sequences, with only a few amino acid sites having variation, and the tail (C-terminal) was more conservative than the head-end (N-terminal) (Fig. 6).

In addition, we also analyzed sequence variations of other flagellar proteins, including extramembrane flagellar structural protein: filament cap protein (encoded by *fliD* gene) (Fig. S8A), junction protein (encoded by *flgK* and *flgL* genes) (Fig. S8B and C), hook cap protein (encoded by *flgD* gene) (Fig. S8D), hook structure protein (encoded by *flgE* gene) (Fig. S8E); intramembrane protein: flagellar rod structure protein (encoded by *flhO* gene) (Fig. S8F). The analysis results showed that the primary sequence homology of these proteins was high (Fig. S8B to F), and only the individual amino acid residues of filament cap protein differed (Fig. S8A). The results above indicate that these structures of the flagella of the intraspecies strains of B. velezensis were all extremely conservative, with little variation.

10.1128/msystems.00778-22.8 FIG S8 Sequence logos of other flagellar proteins. These flagellar proteins included filament cap protein (encoded by *fliD* gene) (A), junction protein (encoded by *flgK* [B] and *flgL* [C] genes), hook cap protein (encoded by *flgD* gene) (D), hook structure protein (encoded by *flgE* gene) (E); and flagellar rod structure protein (encoded by *flhO* gene) (F). These protein sequences were quite conservative, with only a few sites of variation. These sequences came from 190 *B. velezensis* strains in the NCBI database, were aligned using the L-INS-I method of MAFFT (v7.487), and displayed using WebLogo (v.3.7.4). Download FIG S8, JPG file, 2.3 MB. Copyright © 2022 Liu et al. 2022 Liu et al. https://creativecommons.org/licenses/by/4.0/ This content is distributed under the terms of the Creative Commons Attribution 4.0 International license.

In summary, the specific area of flagellin has large variation within B. velezensis strains and the variation degree of flagellin is higher than that of other structural proteins of flagella, whether the N-terminal conserved region or the central variable region, indicating that flagellin evolved faster than other structural proteins of flagella in B. velezensis.

## DISCUSSION

In the natural environment, bacteria will use a variety of methods to maximize the benefit of progeny, and kin discrimination is one of them (21). In this study, we investigated whether flagella were involved in kin recognition of B. velezensis strains. To clarify the role that flagella play in kin recognition, we performed an exchange experiment of kin and non-kin strain’s flagellin on B. velezensis SQR9, then detected their swarm phenotype. Our results showed that flagellin heterogeneity (from kin or non-kin strains) affected recognition of B. velezensis strains.

The *hag* gene encodes *Bacillus* flagellin, and its absence makes SQR9 unable to synthesize flagellar filaments (39). Electron micrographs showed that mutant Δ*hag* has no flagella (Fig. 1B). Strains cannot swarm without flagella (Fig. 1E) but can slide, a short-distance migration movement that does not depend on flagella (40), which accounts for the ability of the Δ*hag* community to spread outward from the inoculation site (Fig. 1E and G). When SQR9wt with flagellar filaments encounter mutant Δ*hag* without flagellar filaments, it will detour (Fig. 1G), instead of the same strain as kin to merge (10), which implied that the lack of flagella filaments changed inherent patterns of communication and cooperation between the 2 populations.

The next stage of investigation revolves around 20 strains of B. velezensis, and the strains with farther kinship distance from SQR9 had wider swarm boundaries with SQR9 (Fig. 2 and Fig. S1), which was consistent with previous reports (13, 33). Here, we need to note that we used B. velezensis as a model given the numerous strains that have been collected. Many plant-growth promoting strains (Bacillus amyloliquefaciens, among others) were reclassified as B. velezensis (41). Then, flagellin of 4 strains (SQR9, FZB42, NB20, and ACCC02961) with different kinship distances from SQR9 was swapped to SQR9, and the results showed that the swarming phenotype between SQR9wt and SQR9 with heterologous flagellin filaments was related to the flagellin divergence of the 2 strains (Fig. 3G to J). The *hag* mutant strain of SQR9 assembling flagellin from its kin merged with wild type when they encountered each other on swarming plates; however, the *hag* mutant strain of SQR9 assembling flagellin of non-kin strains (NB20 and ACCC02961) forms boundary with wild type. Flagellin region interchange experiments further confirmed that flagella are involved in the kin recognition in B. velezensis SQR9.

The execution of outer membrane exchange in M. xanthus requires the initial recognition of 2 cell surface proteins, TraA and TraB, and successful outer membrane exchange can only be achieved when they both are present and have the same or similar structures (42, 43). Some strains of α-, β-, and gammaproteobacteria use the CDI (contact-dependent inhibition) system to secrete adhesins for primary recognition with surface receptors of neighboring cells (44, 45). In our study, flagellin played a role in the kin recognition of B. velezensis SQR9. However, the same SQR9-body means that they have the same virulence and immune system (11), therefore even if they are identified as non-kin as the difference in flagellin, they cannot attack and kill each other.

Given this, we analyzed the differences between the *hag* gene and flagellin structure between kin and non-kin strains. The results showed that the central region length of *hag* gene sequences of kin and non-kin strains varied greatly, ranging from 52 aa to 57 aa (Fig. 5A), which encodes the β-sheet and coils on the D2 domains of the flagellin monomer. Research has shown that the D2 domains contribute to the stability of flagellar filaments and that deletion of the domain also affects the primary anti-flagellin responses (46). In the study, the 4 *Bacillus* strains of flagellin did not contain the D3 domain and had 2 types of flagellin: without D2 domain (SQR9 and FZB42), containing D2 domain (NB20 and ACCC02961) (Fig. 5B to I). The lengths of amino acid sequences of flagellin vary widely among bacteria, especially the variable region at the center, ranging from 8 amino acid residues in Clostridium tetani to 247 amino acid residues in Helicobacter pylori (29). This part of the structure does not participate in the polymerization of flagellin monomers. After the flagellin monomer is assembled into filaments, the structure will be exposed on the periphery of the filaments and directly in contact with the outside environment (29, 47). The wide variation in flagellin structures within species makes them potentially capable of mediating bacterial-bacterial recognition.

Our results showed that flagellin heterogeneity (from kin or non-kin strains) affected recognition of B. velezensis strains. (Fig. 5). Such high variability and rapid evolution of flagellin were reported to exist in many species (48, 49), including 2 levels: the first level is the large difference in flagellin variable regions, which may involve gene transfer across phyla; the second level is that there are evolutionary variations in species, similar to the evolution of other conserved genes (49). For *Bacillus*, sequence diversity of flagellin within the same species has been widely investigated. In Bacillus cereus and Bacillus thuringiensis, flagellin sequence variation is one of the bases for classification (48). Here we found that flagellin sequence variation may plays an important role in kin recognition between B. velezensis strains. These indicate that the *hag* gene has a stronger response to environmental pressure. This intraspecific flagellin differential phenotype is likely to be a differential expression of intraspecific species communication.

In conclusion, our research showed that the flagellin contributes to the kin recognition between B. velezensis strains. However, the specific identification mechanism remains to be explored. In follow-up experiments, it would be interesting to investigate further whether the communication mechanism is flagellar-flagellar or flagellin-receptor specific in *Bacillus* spp.

## MATERIALS AND METHODS

### Strains information and cultural condition.

A list of strains in the study can be found in Table 1, including 20 strains belonging to B. velezensis and several mutants of SQR9 (the strain accession number is 5808 in the China General Microbiology Culture Collection Center, CGMCC, and the genome accession number CP006890 in the National Center for Biotechnology Information [NCBI]). All strains came from Laboratory stock or were isolated from soil (Table 1). All strains were grown at 37°C in low-salt LB (LLB) medium, including 10 g of Tryptone, 5 g of yeast extract, and 3 g of NaCl per L.

**TABLE 1:** Microorganisms used in this study

Strains	Genotype	Reference or source
*B. velezensis* SQR9	Wild type	60
*B. velezensis* FZB42	Wild type	61
*B. velezensis* CC10	Wild type	This study
*B. velezensis* TLi20	Wild type	This study
*B. velezensis* LN1	Wild type	This study
*B. velezensis* b10	Wild type	This study
*B. velezensis* DY24	Wild type	This study
*B. velezensis* RZ17	Wild type	This study
*B. velezensis* SXL34	Wild type	This study
*B. velezensis* CF14	Wild type	This study
*B. velezensis* NJ21	Wild type	This study
*B. velezensis* GZL15	Wild type	This study
*B. velezensis* RZ28	Wild type	This study
*B. velezensis* LYi1	Wild type	This study
*B. velezensis* TLi16	Wild type	This study
*B. velezensis* NB20	Wild type	This study
*B. velezensis* NJ29	Wild type	This study
*B. velezensis* KF29	Wild type	This study
*B. velezensis* YX17	Wild type	This study
*B. velezensis* ACCC02961	Wild type	This study
*B. velezensis* SQR9--pUBXC	*B. velezensis* SQR9 with pUBXC, Zeo^R^	62
Δ*hag*	Mutant of *B. velezensis* SQR9, *hag::Em* (Zeo^R^ Spc^R^)	This study
Δ*bmy*	Mutant of *B. velezensis* SQR9, Δ*bmyD-C* (Zeo^R^)	
Δ*fen*	Mutant of *B. velezensis* SQR9, Δ*fenA-E* (Zeo^R^)	
Δ*dhb*	Mutant of *B. velezensis* SQR9, Δ*dhbA-F* (Zeo^R^)	
Δ*bae*	Mutant of *B. velezensis* SQR9, Δ*baeB-R* (Zeo^R^)	63
Δ*dfn*	Mutant of *B. velezensis* SQR9, Δ*dfnA-M* (Zeo^R^)	
Δ*mln*	Mutant of *B. velezensis* SQR9, Δ*mlnA-I* (Zeo^R^)	
Δ*srf*	Mutant of *B. velezensis* SQR9, Δ*srfAA-AD* (Zeo^R^)	
ΔGI	Mutant of bacillunoic acid synthesis gene island *in B. velezensis* SQR9, *GI::Cm* (Zeo^R^ Cm^R^)	36
Δ*hag-hag*_SQR9_	*B. velezensis* SQR9, *hag::Spc*, pNW33N-*hag*_SQR9_ (Zeo^R^ Spc^R^ Cm^R^)	This study
Δ*hag-hag*_FZB42_	*B. velezensis* SQR9, *hag::Spc*, pNW33N-*hag*_FZB42_ (Zeo^R^ Spc^R^ Cm^R^)	This study
Δ*hag-hag*_NB20_	*B. velezensis* SQR9, *hag::Spc*, pNW33N-*hag*_NB20_ (Zeo^R^ Spc^R^ Cm^R^)	This study
Δ*hag-hag*_ACCC02961_	*B. velezensis* SQR9, *hag::Spc*, pNW33N-*hag*_ACCC02961_ (Zeo^R^ Spc^R^ Cm^R^)	This study

### Construction of mutants and flagellin gene complementary strains of B. velezensis SQR9.

To delete the *hag* gene in the SQR9 genome, the upstream and downstream regions (1039 bp and 973 bp) that flanked the *hag* gene were amplified from the SQR9 genome, and the primers used to amplify upstream and downstream regions were: up-F (5′-CTCGTCGACATTGACTGCATT-3′) and up-R (5′- CGTTACGTTATTAGTTATGCTAGTGTTAAGAGCCGCGAT-3′), down-F (5′-TATAGCATACATTATACGTGCGCAAGCTAACCAACAGC-3′) and down-R (5′-CGGCATTGGCCGTCAGTTCA-3′). The spectinomycin (Spc) resistance gene was amplified from plasmid P7S6 using the primers spc-F (5′-ATCGCGGCTCTTAACACTAGCATAACTAATAACGTAACG-3′) and spc-R (5′-GCTGTTGGTTAGCTTGCGCACGTATAATGTATGCTATA-3′), and the amplified regions (1029 bp) contained 18 bp overlap with the upstream and downstream fragments of the *hag* gene, respectively. The mixture volume (50 μL) for amplifying upstream, downstream, and Spc resistance gene fragments was: 18 μL of water, 1 μL of 1 × Phanta Max Master Mix DNA polymerase (Vazyme), 1 μL of dNTP mix, 25 μL of Buffer, 2 μL of the forward primer, 2 μL of the reverse primer, and 1 μL of DNA template. The PCR program was performed under the following conditions: 98°C for 2 min and 32 cycles at 98°C for 10 s, 55°C for 10 s, and 72°C for 4 min.

Upstream, downstream, and *spc* resistance gene fragments were fused using the method of two-step overlapping PCR (50). The mixture volume (25 μL) for the first step was: 5 μL of water, 0.5 μL of 1 × Phanta Max Master Mix DNA polymerase (Vazyme), 1 μL of dNTP mix, 12.5 μL of Buffer, 2 μL (100 ng) of the upstream fragment, 2 μL (100 ng) of downstream fragment, and 2 μL (100 ng) of resistance gene fragment. The PCR program was performed under the following conditions: 98°C for 2 min and 12 cycles at 98°C for 10 s, 50°C for 10 s, and 72°C for 4 min. For the second step, the mixture volume (50 μL) contained 18 μL of water, 1 μL of 1 × Phanta Max Master Mix DNA polymerase (Vazyme), 1 μL of dNTP mix, 25 μL of Buffer, 2 μL of the primer up-F (the forward primer of the upstream fragment), 2 μL of the primer up-R (the reverse primer of the downstream fragment), and 1 μL of product from the first PCR step. In addition, the PCR program was performed under the following conditions: 98°C for 2 min and 32 cycles at 98°C for 10 s, 55°C for 10 s, and 72°C for 4 min.

After purification of the fused fragment of 3 genes (upstream, downstream, and Spc resistance gene), the transformation was conducted by the artificial induction of genetic competence. When SQR9 with plasmid pUBXC (carrying the xylose-inducible comK expression cassette) was cultivated to an OD_600_ of 0.5 in LB medium, 1% (wt/vol) xylose was added. After 1 h of incubation, 20 μL fused fragment was mixed with 200 μL SQR9 cells in a 2 mL centrifuge tube and incubated at 37°C for 7 h. Then, cells were plated on LB agar plates including 100 μg mL^−1^ Spc, and the correct mutants were verified by sequencing (51).

To obtain 4 flagellin gene complemented strains B. velezensis SQR9, the *hag* gene of these strains: SQR9, FZB42, NB20, and ACCC02961 were amplified with primers *hag* (F:5′-GCTCTAGAGAAGCGCCTCAGCACGTAGA-3′, R:5′-CGCGGATCCGAGAACCAGGGATCTTTCCGTC-3′) containing 2 cleavage site of restriction endonuclease (XbaI: TCTAGA and BamHI: GGATCC), and the amplification of the *hag* gene fragments was the same as above. Then, the obtained fragments were ligated into the plasmid pNW33N (52) using restriction enzyme ligation technology.

The *hag* gene fragments and plasmid pNW33N (carry chloramphenicol [Cm] resistance gene) were digested with restriction endonucleases: XbaI and BamHI (TaKaRa), and the reaction system and conditions were referred to the instructions on the website of the TaKaRa bio (https://www.takarabiomed.com.cn/Product.aspx?m=20150106133447710028). After that, the *hag* gene fragment with the 2 sticky ends exposed and the plasmid pNW33N was ligated overnight at 16°C, and the enzymatic ligation system was as follows (10 μL): 5 μL Solution I (TaKaRa), 1 μL (100 ng) plasmid pNW33N and 4 μL (400 ng) *hag* gene fragment. The ligation mixture was transferred into Δ*hag* mutant with plasmid pUBXC using the xylose induction method mentioned above. Then, cells were plated on LB agar plates including 100 μg mL^−1^ Spc and 5 μg mL^−1 ^Cm, and the correct complement strains were verified by extracting plasmid and sequencing. All these mutants and flagellin gene complementary strains are listed in Table 1.

### Swarm assay.

Swarm assays were performed on the 9 cm plates containing B-medium with 0.7% agar at 37°C (13). Strains were grown on solid LLB plates at 37°C for 12 h before use and then transferred to 3 mL of liquid B-medium and shaken overnight at 37°C. The overnight cultures were then diluted to an optical density (OD_600_) of 0.5, and 2 μL was spotted on the agar plates. The plates with a cover were dried in a laminar flow hood for 30 min, sealed and incubated for 2 days at 37°C, and photographed. Regarding the determination of boundary widths of non-kin strains in swarm boundary assays, each pair of strains included 6 replicates of the swarm boundary phenotype, and each replicate was measured three times using the Image J (v.1.53c) (53). The point plots were drawn using the ggplot2 package and linear regression analysis is performed using the lm() function in R (v.4.0.3).

### Motility test.

To test the motility ability of different strains, 3 kinds of B-medium with 0.3%, 0.5%, and 0.7% agar were selected. The strain preparation and culture conditions were the same as above. Pictures of the plates were acquired 24 h after inoculation. The halo area of the strains was measured in Image J (v.1.53c).

### Determination of surfactin production.

Forty milliliters of sterile supernatant of the tested strains were cultured in Landy medium (54) at 30°C for 60 h, the pH was adjusted to 2.0 with 6 mM HCl, and sat at 4°C overnight. It was then centrifuged to retain the pellet, 4 mL of methanol (LC/MS, Merck) was added to soak for 5 h, and the sample was filtered through a 0.22 μm membrane to obtain a sterile crude extract, which was stored at 4°C for testing.

Determination of surfactin production of the strains was performed using an HPLC 1200 apparatus (1200 series; Agilent). A high performance liquid chromatography (HPLC) system equipped with an Agilent ZORBAX Eclipse XDB-C18 (250 × 4.6 mm, 5 μm) column was operated and maintained at 30°C. A mobile phase mixture consisting of an Acetonitrile and 0.1% (vol/vol) CH_3_COOH solution (ratio of 88:12) was pumped in an isocratic mode with a flow rate of 0.84 mL min^−1^. The injection volume of the sample was set at 20 μL and was detected through a VWD detector at 210 nm. Each analysis was completed within 20 min.

Surfactin standard solution (1000 mg L^−1^) was prepared from 99% pure surfactin (shyuanye). The surfactin substance peaks in the sample to be tested were determined by comparing the chromatographic peak of the sample with the surfactin standard solution, and the yield of surfactin in the sample was characterized by the sum of the absorbance values of the last 3 well-separated surfactin substance peaks. Each sample was tested in triplicate. Analysis of significant differences was performed using independent sample T-Test (*P < *0.05) on SPSS (v. 25).

### Yield determination of bacillunoic acids.

The bacillunoic acid production of strains SQR9, Δ*hag*, Δ*hag-hag*_SQR9_, Δ*hag-hag*_FZB42_. Δ*hag-hag*_NB20_, and Δ*hag-hag*_ACCC02961_ were evaluated by the inhibitory effect of the fermentation supernatant on the target strain B. velezensis FZB42.

Five milliliters of a diluted overnight culture of FZB42 (~10^5^ CFU mL^−1^) was spread onto LLB plates (10 × 10 cm) to be grown as a bacterial lawn. The supernatant of the strain to be tested that was cultured in medium B for 48 h (37°C, 170 rpm), was concentrated 3 times using a centrifugal filter (10 kDa, Amicon Ultra-15), then 180 μL was added to the Oxford cup on the bacterial lawn, and then the plate was placed at 22°C until a clear zone formed around the Oxford cup. It was then photographed, and the antagonistic circle was measured. The antagonism assays for each strain included 9 replicates. The box plots were drawn using R (v.4.0.3).

### PCR amplification.

Genomic DNA was extracted using omega Bacterial DNA Kit D3350 (Omega, Bio-tek), and the concentration and quality of DNA were assessed using a NanoDrop 2000 spectrophotometer. The *gyrA* and *hag* gene sequences of 20 B. velezensis strains were amplified by PCR with primers *gyrA* (F:5′-CAGTCAGGAAATGCGTACGTCCTT-3′, R:5′-GTATCCGTTGTGCGTCAGAGTAAC-3′) and *hag* (F:5′-AGAGTTTGATCCTGGCTCAG-3′, R:5′-GGTTACCTTGTTACGACTT-3′), and were subsequently Sanger sequenced. The sequences of *gyrA* and flagellin protein of 20 B. velezensis strains were in Dataset S1 at [https://zenodo.org/record/7131368#.YzeloNhBxPY] and Dataset S2 at [https://zenodo.org/record/7131381#.YzemJdhBxPY], respectively.

### Phylogenetic analysis.

In this study, the phylogenetic analysis of genes was conducted using MEGA (v.5.05) for Neighbor-Joining (55). The 1000 bootstrap replications tested the clades' reliability. Furthermore, annotation and beautification of trees were achieved through the iTol online site (https://itol.embl.de) (56).

### Electron microscopy.

We cultivated the strains on the LLB solid medium at 37°C for 8 h, placed the plate at an angle, and soaked the fresh colony in sterile deionized water for 2 h, during which we gently shook the plate every 20 min. Then, the strain suspension on the copper net was air dried, the flagella of strains were observed with a HT7700 transmission electron microscope (TEM) that operated at 80 kV, and photographed.

### Protein structure prediction.

The *hag* and *fliD* completed sequences gene of strains SQR9, FZB42, NB20, and ACCC02961 were obtained from the NCBI genome database (SQR9 and FZB42) and sequenced draft genomes (NB20 and ACCC02961). The protein tertiary structure of flagellin monomer, flagellin homomer, and flagellar cap were predicted on the Swiss-Model website (https://swissmodel.expasy.org/).

### Protein sequences analysis of flagellar proteins.

In total, 395 available genomes of B. velezensis were downloaded from the NCBI database using the ncbi-genome-download script (https://github.com/kblin/ncbi-genome-download/) (Table S2A, see [https://zenodo.org/record/7131360#.YzelJ9hBxPY]). The complete genomes were kept, and genomes of the whole genome shotgun were filtered out. Then, the location information of genes (*hag*, *fliD*, *flgK*, *flgL*, *flgD*, *flgE*, and *flhO*) sequences on genomes were obtained by alignment with corresponding genes sequences of SQR9 using NCBI-blast+ (v.2.9.0), and the genes sequences were extracted using Fasta Extract tool in TBtools (v1.0986853) (57). Finally, the 190 genomes of B. velezensis that have whole genes were retained and translated into protein sequences using the online tools of the EMBL website (https://www.ebi.ac.uk/Tools/st/emboss_transeq/) for the next analysis (Table S2B, at [https://zenodo.org/record/7131360#.YzelJ9hBxPY]).

These protein sequences of genes were aligned using the L-INS-I method of MAFFT (v7.487) (https://mafft.cbrc.jp/alignment/software/), and the protein sequences variation of genes were displayed using WebLogo (v.3.7.4) (http://weblogo.threeplusone.com/) (58) and Jalview (v.2.11.1.5) (https://www.jalview.org/) (59).

### Statistics.

Duncan's multiple range tests (*P < *0.05) of the SPSS version 25.0 (IBM, Chicago, IL, version 25.0) was used for statistical analysis of differences among treatments.

### Data availability.

The accession numbers of the genome sequence of B. velezensis SQR9, FZB42, NB20 and ACCC02961 in the NCBI are: CP006890, NC_009725.2, JALJAJ000000000, and JALJAK000000000.

## Supplementary Material

Reviewer comments
