# Supplementary figures and images for: Involvement of Flagellin in Kin Recognition between Bacillus velezensis Strains
Source: mSystems. 2022 Oct 11;7(6):e00778-22. doi: 10.1128/msystems.00778-22 (PMC9764977; doi:10.1128/msystems.00778-22)

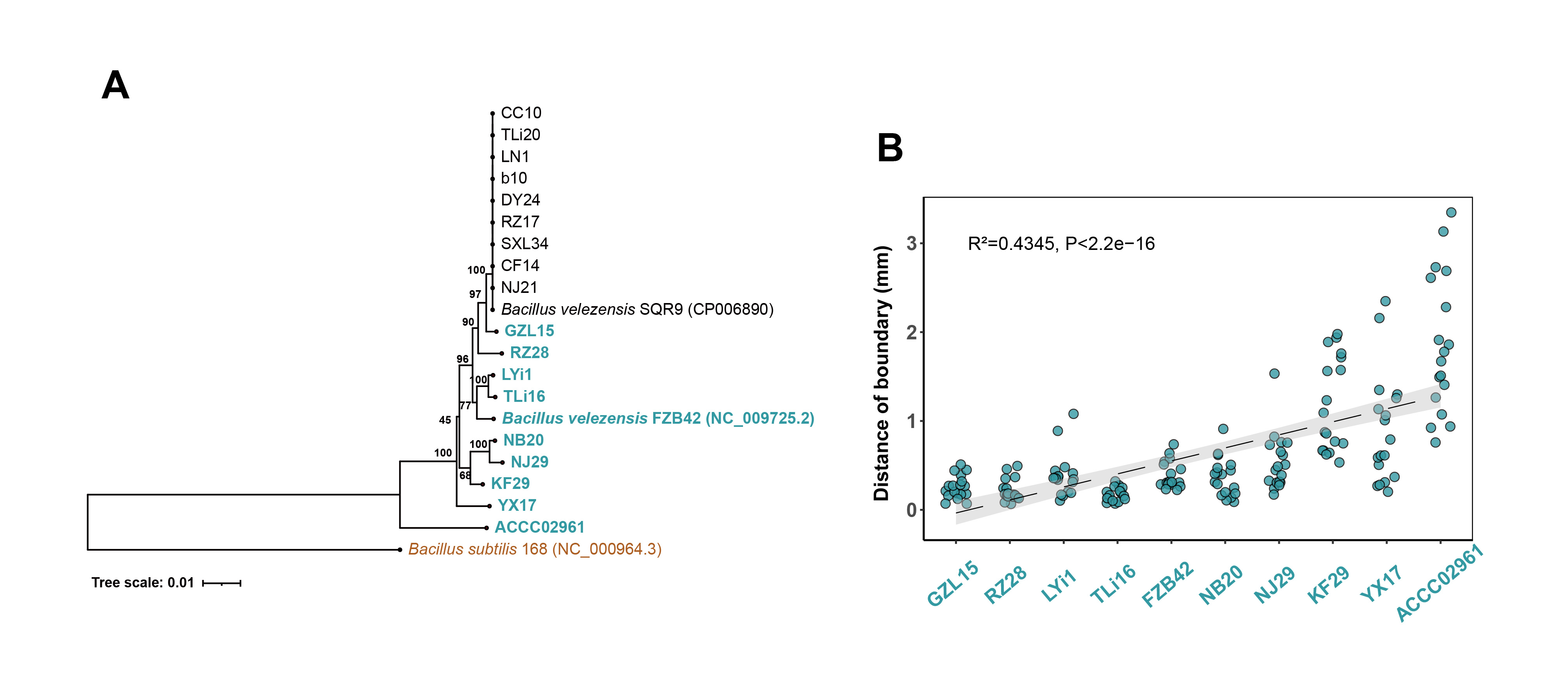

Supplement: FIG S1 [file msystems.00778-22-s0001.jpg]

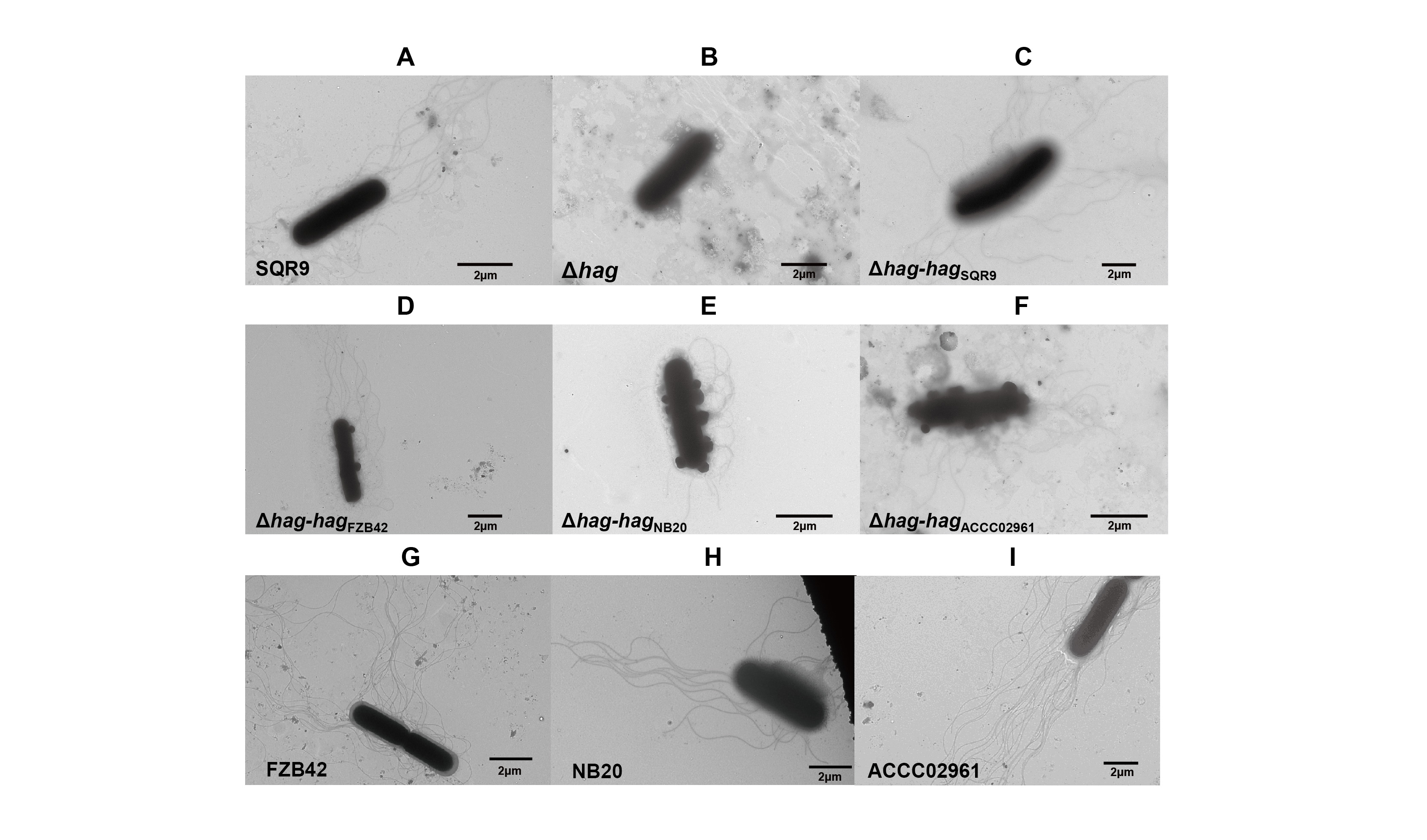

Supplement: FIG S2 [file msystems.00778-22-s0002.jpg]

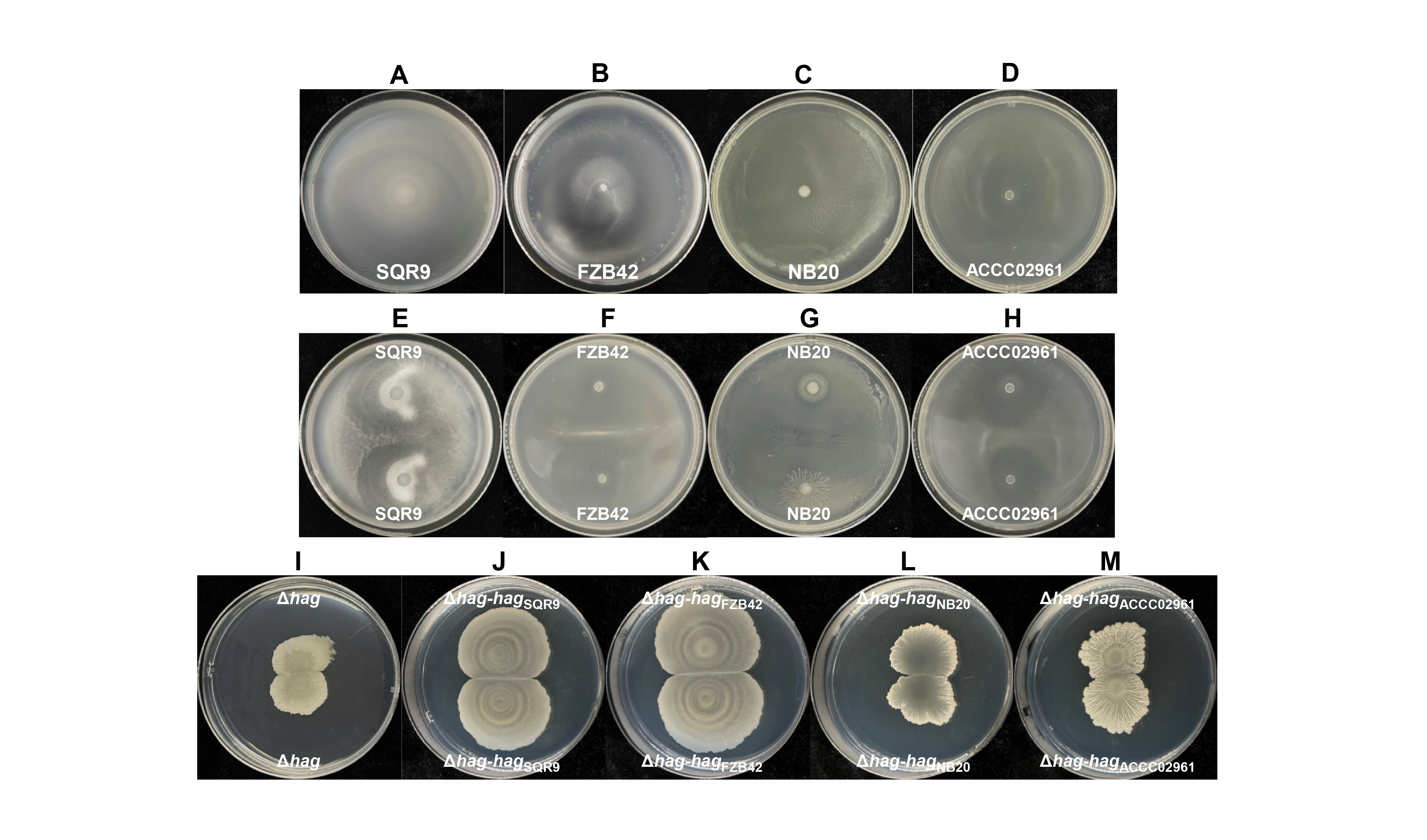

Supplement: FIG S3 [file msystems.00778-22-s0003.jpg]

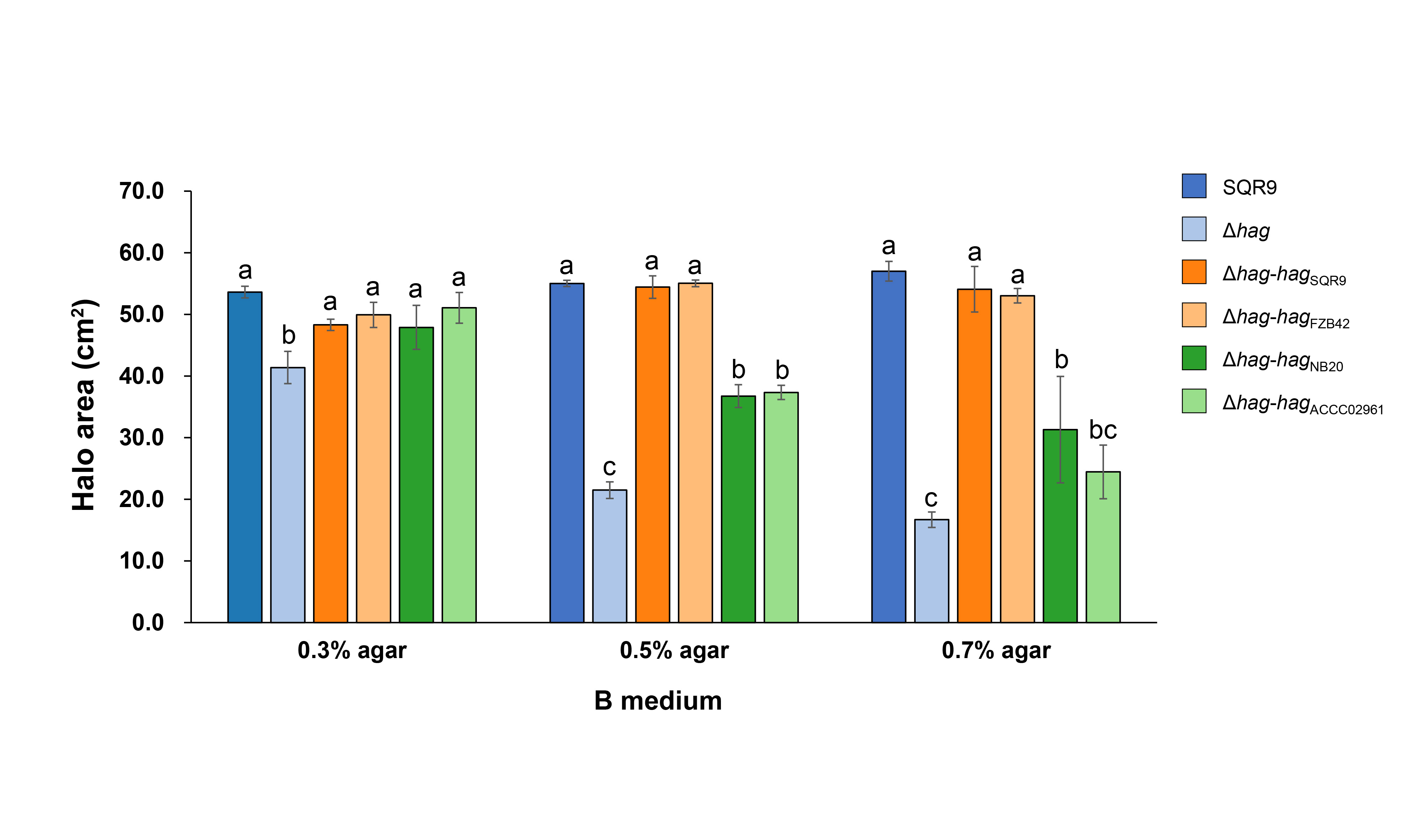

Supplement: FIG S4 [file msystems.00778-22-s0004.jpg]

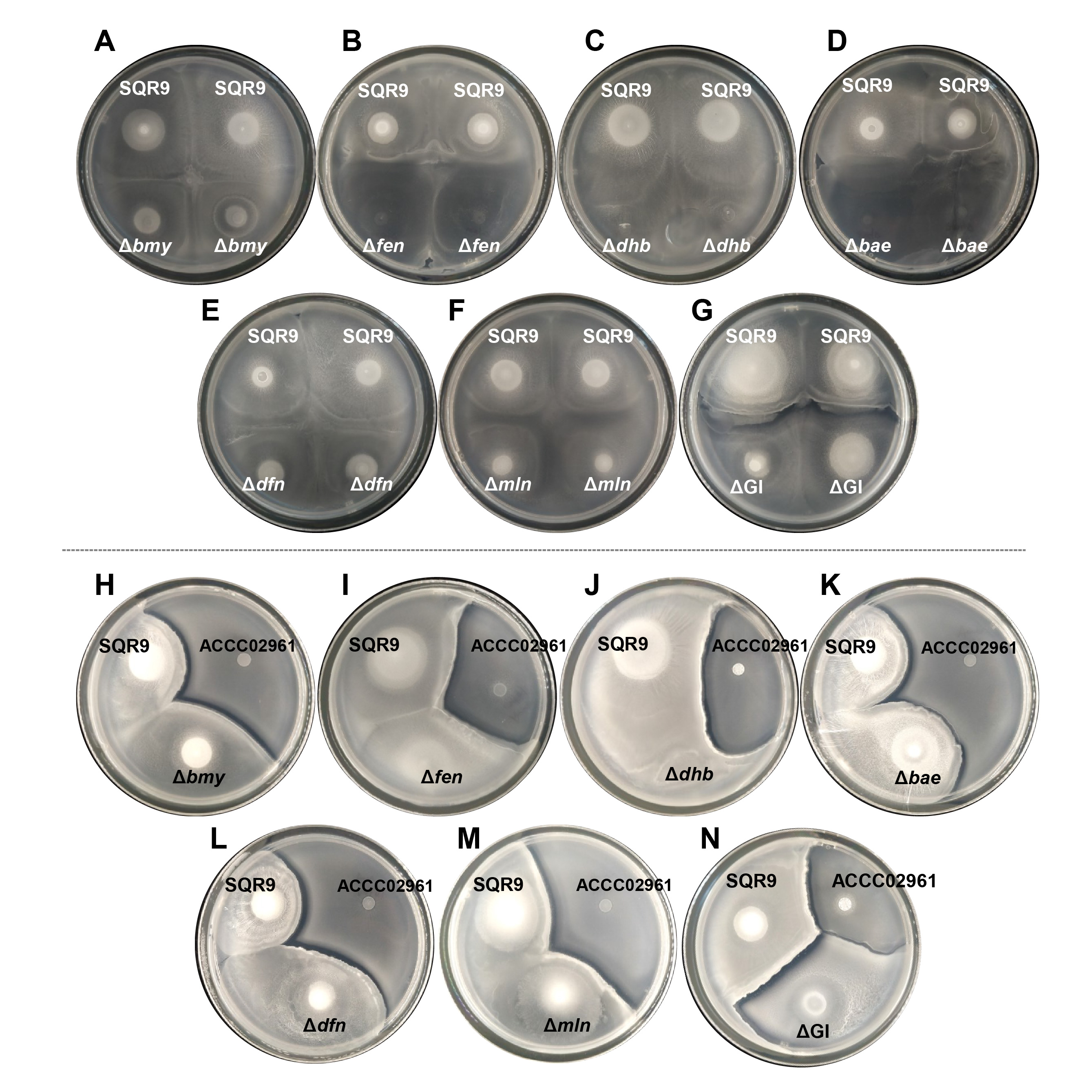

Supplement: FIG S5 [file msystems.00778-22-s0005.jpg]

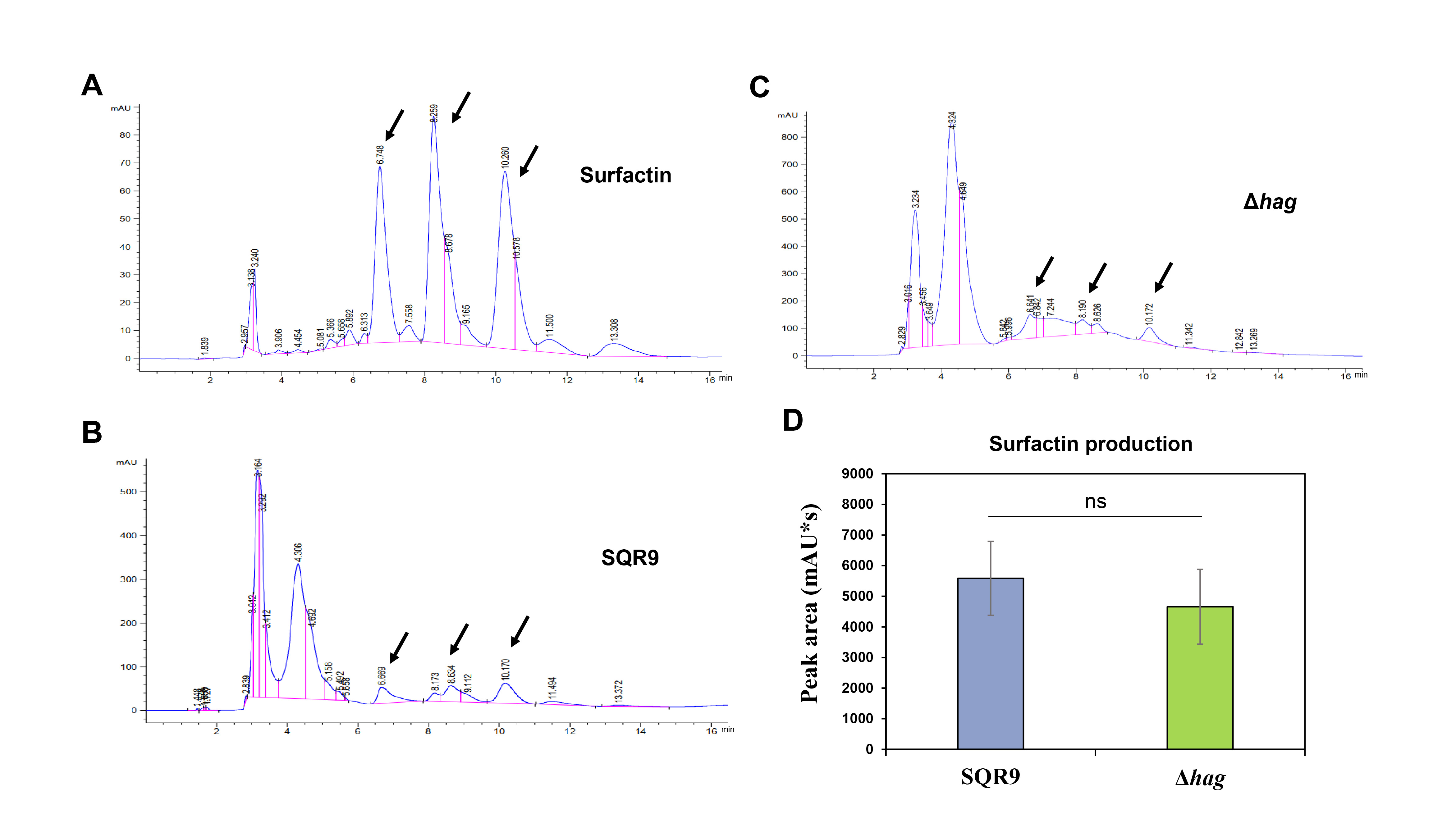

Supplement: FIG S6 [file msystems.00778-22-s0006.jpg]

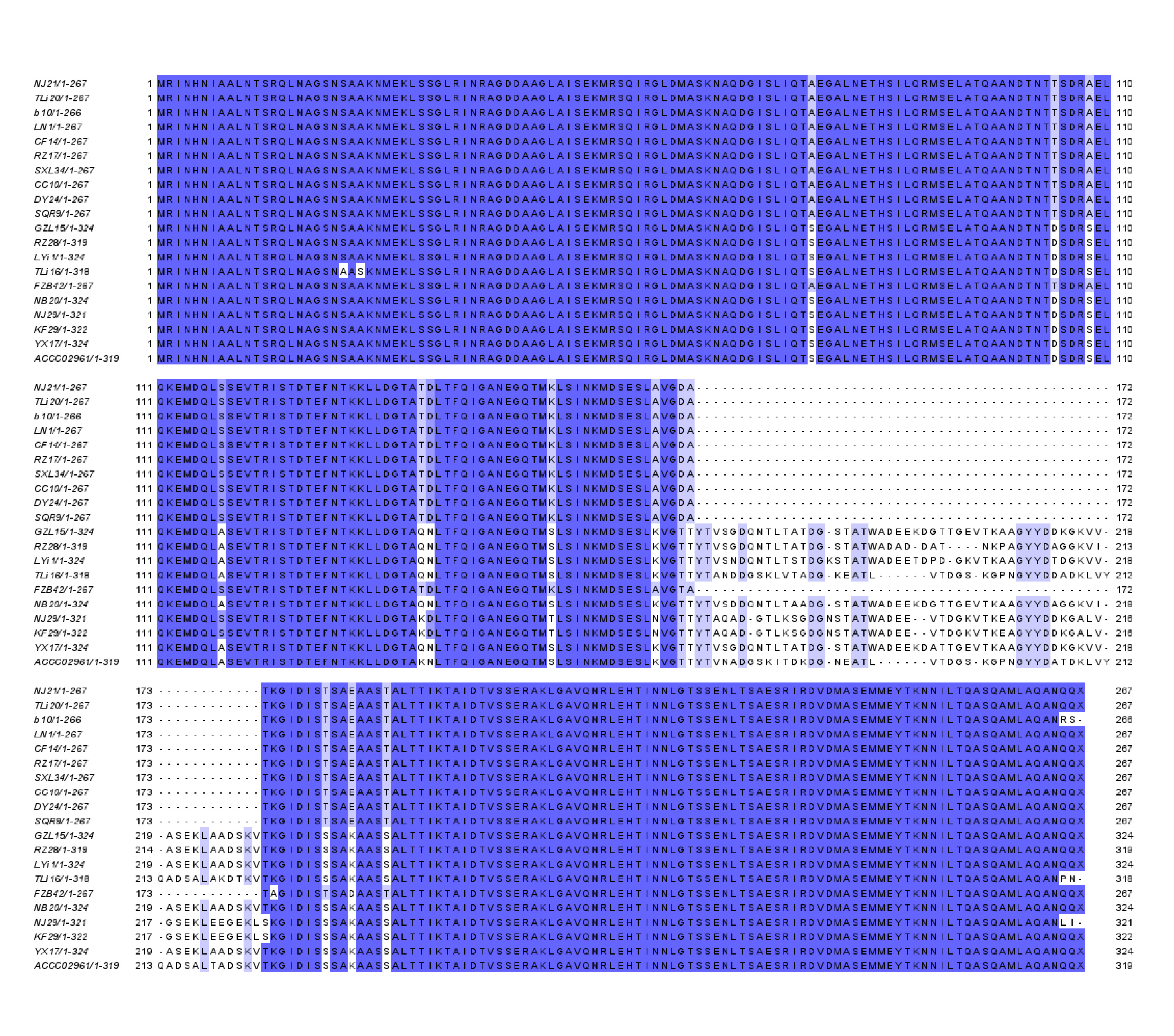

Supplement: FIG S7 [file msystems.00778-22-s0007.jpg]

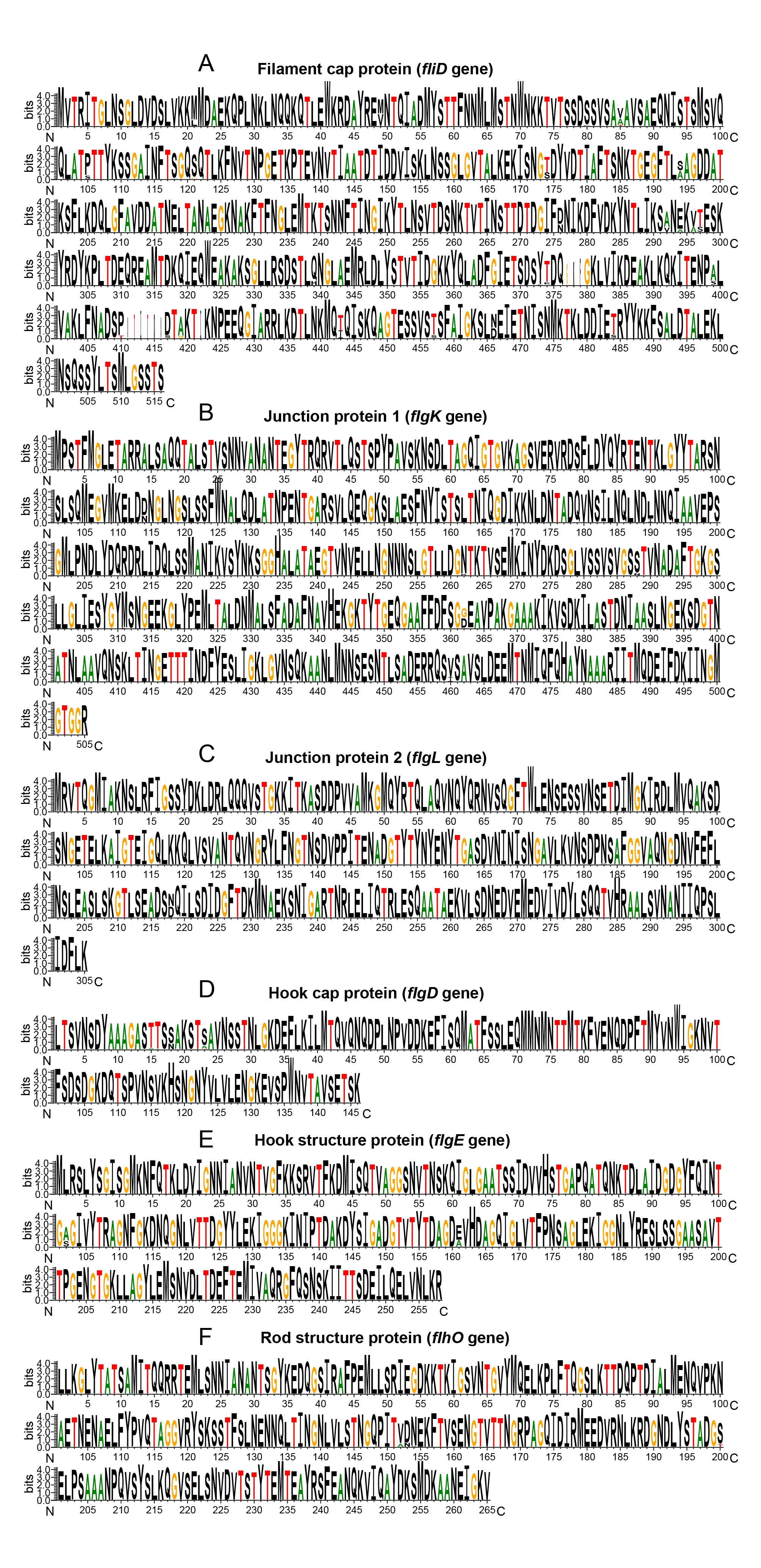

Supplement: FIG S8 [file msystems.00778-22-s0008.jpg]
